# The discovery of *Paratestophis gelicolus* gen. nov., sp. nov. from the rainbow water snake, *Enhydris enhydris*, in Thailand, with systematic update of Echinochasmidae Odhner, 1910

**DOI:** 10.1017/S0031182025100863

**Published:** 2025-11

**Authors:** Vachirapong Charoennitiwat, Sila Viriyautsahakul, Abigail Hui En Chan, Kittipong Chaisiri, Supakit Tongpon, Panithi Laoungbua, Tanapong Tawan, Taksa Vasaruchapong, Urusa Thaenkham, Napat Ratnarathorn

**Affiliations:** 1 Department of Helminthology, Faculty of Tropical Medicine, Mahidol University, Bangkok, Thailand; 2 Applied Animal Science Laboratory, Department of Biology, Faculty of Science, Mahidol University, Bangkok, Thailand; 3 The Thai Red Cross Society, Snake Farm, Queen Saovabha Memorial Institute, Bangkok, Thailand

**Keywords:** molecular identification, morphology, new genus, new species, *Paratestophis*, rainbow water snake, snake parasite

## Abstract

A new genus and species of trematode, *Paratestophis gelicolus* gen. nov., sp. nov., is described from the large intestine of the rainbow water snake, *Enhydris enhydris*, collected from several provinces in southern Thailand. Morphological analyses reveal distinct characteristics that differentiate *P. gelicolus* gen. nov., sp. nov. from related echinochasmid taxa, specifically its elongated bottle-shaped body, presence of 22 collar spines, parallel testes and parasitism of snakes–features not observed in other echinochasmid genera. Multi-marker phylogenetic analyses (28S rRNA, 18S rRNA, ITS2 and *COI*) strongly support its taxonomic placement within Echinochasmidae while confirming its genetic distinction from known genera such as *Echinochasmus, Stephanoprora*, and *Microparyphium*, thereby warranting the establishment of *Paratestophis* gen. nov. The species exhibited a 24% prevalence of infection (25/106) in *E. enhydris*, and was found co-infecting with four other helminths, including *Tanqua siamensis, Encyclometra bungara*, and two additional trematode species currently under examination, all occupy distinct ecological niches. Principal Component Analysis based on 19 morphological characters revealed morphological homogeneity among the specimens. This study represents the first record of a new genus and species within Echinochasmidae infecting snakes, and provides an updated systematic framework for the family, including a revised key to genera. The findings emphasise the need for further research into parasite taxonomy, host specificity and evolutionary relationships in Southeast Asian ecosystems.

## Graphical abstract

**Figure:**
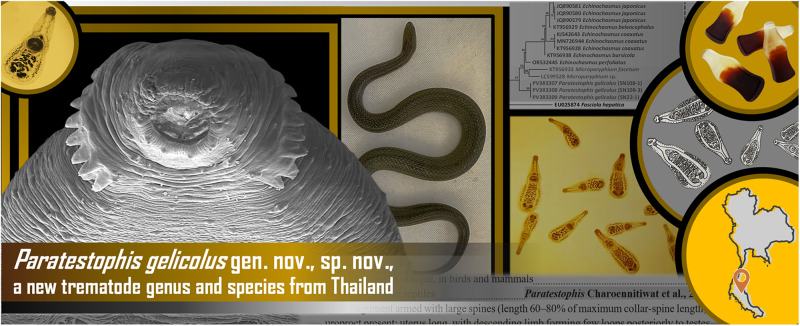

## Introduction

The family Echinochasmidae (Odhner, 1910) consists of globally distributed trematodes that parasitise reptiles, birds and mammals, with reported cases of human infections that can lead to severe gastrointestinal symptoms such as epigastric or abdominal pain, diarrhoea, fatigue and malnutrition (Toledo *et al.*, 2022). Notable species implicated in human infections include *Echinochasmus japonicus* (Sayasone *et al.*, 2009) and *Echinochasmus caninus* (Chai *et al.*, 2019). In reptiles, infections by echinochasmid species have been rarely reported–primarily in crocodiles (Platt, 2006; Cajiao-Mora *et al.*, 2025)–with no previous records of infection in snakes. The family Echinochasmidae exhibits a distinct combination of morphological characteristics, including the absence of a ventral connection ridge on the collar and the presence of collar spines arranged in a dorsally interrupted row, attributed to a mid-dorsal depression on the collar (Kostadinova, 2005; Islas-Ortega *et al.*, 2024).

During our investigation of parasitic helminths in snakes, digeneans were found at a moderate prevalence in the intestinal tracts of the rainbow water snakes (*Enhydris enhydris*) collected from southern Thailand. Although initial molecular analyses classified these specimens within the genus *Echinochasmus* due to their close genetic similarity, their distinct morphological characteristics differed significantly from those of known *Echinochasmus* species and other members of the family Echinochasmidae (*cf.* Jones *et al.*, 2005), prompting a detailed taxonomic analysis using multiple approaches. Subsequent biological and systematic analyses confirmed that these specimens represent a novel genus and the first described species within this genus, which are formally introduced in this present study. Additionally, the systematic framework of the newly established genus within Echinochasmidae is updated herein.

Systematic studies on Echinochasmidae have undergone periodic revisions. Initially classified as the subfamily Echinochasminae (Jones *et al.*, 2005), it was later redescribed and elevated to a separate family within the superfamily Echinostomatoidea Looss, 1902 (Tkach *et al.*, 2016). The family currently comprises seven genera: *Echinochasmus* Dietz, 1909; *Dissurus* Verma, 1936; *Mehrastomum* Saksena, 1959; *Microparyphium* Dietz, 1909; *Pulchrosomoides* Freitas & Lent, 1937; *Saakotrema* Skrjabin & Bashkirova, 1956; and *Stephanoprora* Odhner, 1902 (Tkach *et al.*, 2016; Islas-Ortega *et al.*, 2024). However, the molecular data for some genera remain unstudied, leaving their taxonomic status unresolved and their systematic classification controversial (Tkach *et al.*, 2016; Islas-Ortega *et al.*, 2024).

A substantial number of digenean specimens were examined using a combination of molecular phylogenetic techniques and detailed morphological assessments. The primary objective of the morphological analysis was to identify key diagnostic features of the new genus and species within a broader taxonomic framework, complementing the molecular evidence. Phylogenetic relationships were investigated through the reconstruction of genetic sequences from four genetic markers, including the mitochondrial cytochrome c oxidase subunit 1 (*COI*), the small and large subunits of nuclear ribosomal RNA (18S and 28S rRNAs), and the nuclear internal transcribed spacer 2 region (ITS2). This study aims to provide molecular and morphological evidence to support the description of the newly identified genus and species, *Paratestophis gelicolus* gen. nov., sp. nov., contributing to an update of genera within family Echinochasmidae.

## Materials and methods

### Host and parasite specimen preparation

Rainbow water snakes (*E. enhydris*) were collected by local villagers and rescuers in Nakhon Si Thammarat and nearby provinces in southern Thailand before being transported to the Snake Farm at the Queen Saovabha Memorial Institute (QSMI) in Bangkok. Between 2021 and 2025, 106 snake specimens that perished during quarantine in the farm were dissected and subsequently transferred to the Department of Helminthology, Faculty of Tropical Medicine, Mahidol University, Bangkok, for parasite examination. The snakes were stored at −20 °C until dissection.

Prior to dissection, species of the snakes was identified based on morphological characteristics as described by Cox *et al.* (2012). Dissections were performed following the protocol outlined by Ratnarathorn and Kongrit (2024). The large intestine, which primarily harboured the target parasitic trematodes, was carefully isolated from surrounding organs and placed in Petri dishes containing water to prevent desiccation. The intestine was teared open and examined for *Paratestophis gelicolus* gen. nov., sp. nov. under stereomicroscopes (Olympus SZ30 and SZ51, Japan). When detected, the parasites were gently extracted and preserved in 70% ethanol. Additionally, six complete specimens in excellent conditions were preserved in 2.5% glutaraldehyde solution in 0.1 M sucrose phosphate buffer (SPB) for scanning electron microscopy (SEM) analysis.

### Morphological study

Thirty complete individual parasites (from 12 out of 25 infected hosts) were selected from the 70% ethanol stock for morphological studies and permanent slide preparation. Of these, four specimens were designated as the holotype and paratypes. Specimens from the remaining other 13 infected snakes were unsuitable for measurement due to deformation or incompleteness, with few retained for genetic analysis. Each selected specimen was transferred and fixed in 10% formalin overnight, rinsed in distilled water for 10 minutes, and stained with acetocarmine for 3 hours. Destaining was performed using a 1% acid (HCl)-alcohol solution for 10 seconds, followed by a graded ethanol dehydration series (70%, 80%, 90%, 95%, and 100%), with each step lasting 10 minutes. The specimens were then immersed in a 1:1 100% ethanol:xylene solution for 10 minutes, briefly placed in pure xylene, and subsequently mounted in Permount™ on glass slides with coverslips. The mounted slides were left at room temperature for several days to ensure complete drying.

**Figure 1.**
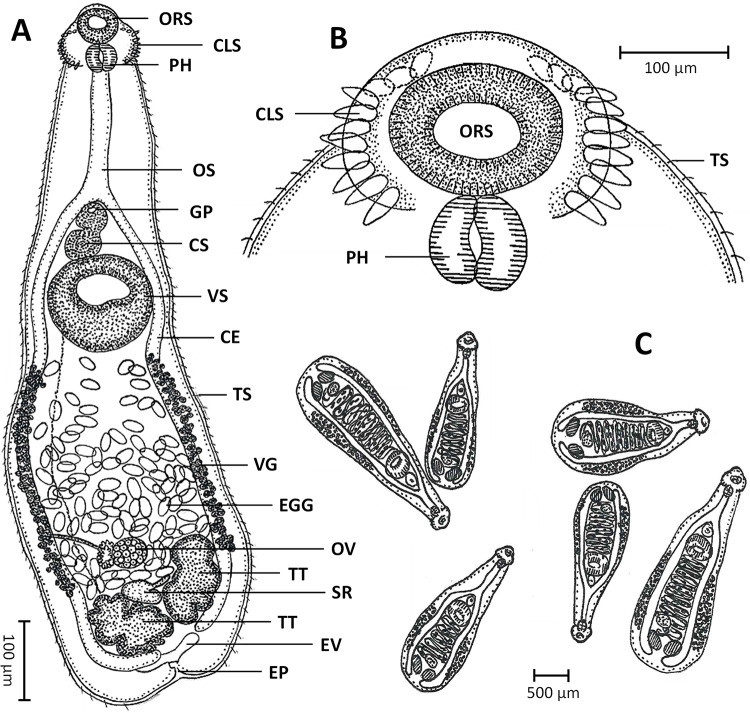
*Paratestophis gelicolus* gen. nov., sp. nov.: (A) Entire body, ventral view; (B) Oral region showing collar spines, ventral view; and (C) Bottle-like body shape of *P. Gelicolus* gen. nov., sp. nov. Observed in the intestine. CE, caecum; CLS, collar spine; CS, cirrus sac; EGG, eggs; EP, excretory pore; EV, excretory vesicle; GP, genital pore; ORS, oral sucker; OS, oesophagus; OV, ovary; PH, pharynx; SR, seminal receptacle; TS, tegumental spine; TT, testis; UT, uterus; VG, vitelline gland; VS, ventral sucker.

A comprehensive examination of parasite morphology was conducted on permanently mounted specimens using ZEN 2 Blue Edition software, connected to an inverted microscope (Zeiss Primovert, Germany) equipped with a Zeiss Axiocam. Illustrations (Figure 1) were created using a light microscope with a camera lucida (Leitz, Wetzlar, Germany). As a newly described genus within the family Echinochasmidae (Odhner, 1910), the taxonomic keys for species identification and relevant morphological features were updated based on Jones *et al.* (2005) and (Islas-Ortega *et al.*, 2024), with *Paratestophis* gen. nov., included in the identification key.

**Figure 2.**
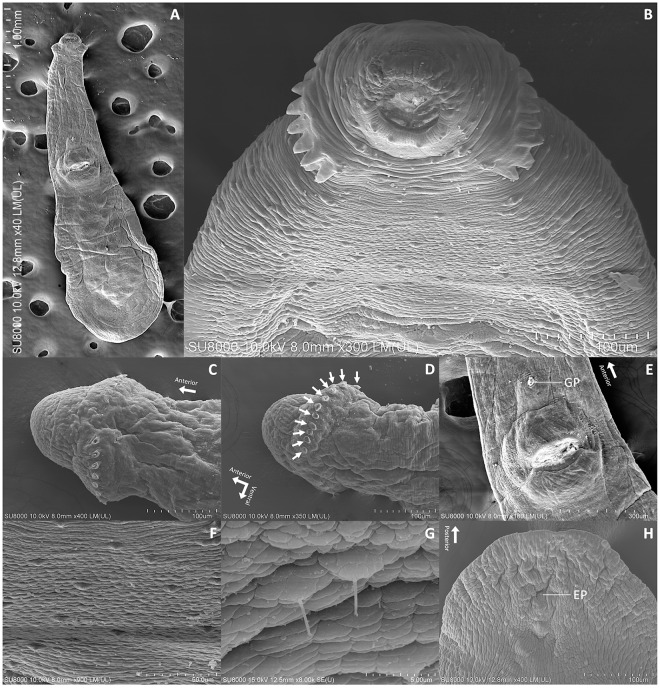
Scanning electron micrographs of *Paratestophis gelicolus* gen. nov., sp. nov.: (A) Entire body, ventral view; (B) Oral sucker with collar spines and randomly distributed dot-like tegumental spines in the anterior region, apical view; (C) Single-row arrangement of collar spines interrupted dorsally in the anterior region, dorsal view; (D) Single-row arrangement of 11 collar spines (white arrows, one side) interrupted ventrally in the anterior region, lateral view; (E) Ventral sucker with the genital pore; (F) Randomly and sparsely distributed tegumental spines; (G) Magnified view of tegumental spines; and (H) Excretory pore in the posterior region. EP, excretory pore; GP, genital pore.

The SEM analysis was conducted on six specimens initially preserved and fixed in 2.5% glutaraldehyde solution. The specimens underwent secondary fixation in 1% osmium tetroxide in 0.1 M SPB, followed by dehydration with ethanol and drying using a critical point dryer (CPD300 auto, Leica, Wetzlar, Germany). After dehydration, the parasites were coated with gold using a sputter coater (Q150R PLUS, Quorum, East Sussex, the UK). The sample preparation process was conducted at the Department of Tropical Pathology, Faculty of Tropical Medicine, Mahidol University, while SEM imaging (SU8010, Hitachi High-Tech, Japan) was performed at the Central Instrument Facility, Faculty of Science (Phaya Thai), Mahidol University (see Figure 2 for illustrations).

To assess morphological variation among the 30 parasite specimens, 19 distinct morphological characters were measured and expressed in micrometres (μm). The measurements are presented as the mean ± standard deviation, with the range provided in parentheses. The measured characteristics included body length, maximum body width, forebody length, head collar length, head collar width, pharynx length, pharynx width, oral sucker length, oral sucker width, cirrus sac length, cirrus sac width, uterus length, ovary length, ovary width, length and width of each testis, post-testicular-field length, egg length, and egg width (see Table 1 and S1). Four of these variables, including maximum body width, forebody length, uterus length, and post-testicular field, were further calculated as percentages of the total body length. In addition, three spine-related characteristics were assessed using SEM images: the number of collar spines, the distribution pattern of tegumental spines, and a relative size comparison between collar and tegumental spines. To fully describe the species, features of the oesophagus, intestinal ceca, vitelline glands, excretory vesicle, internal seminal vesicle, pars prostatica, cirrus, Mehlis’ gland, metraterm, and testis shape and alignment were examined and described from stained specimens with the focal organs visible under Olympus CX31 Biological Microscope (Olympus Corporation, Tokyo, Japan).

**Table 1.** Measurement features for the holotype and all examined specimens of *Paratestophis gelicolus* gen. nov., sp. nov in this study. Measurements are given in micrometres (μm)

Characteristics	Holotype	Range (all specimens, n = 30)	Mean ± SD
Body length (BL)	3432	1653‒3523	2496 ± 545
Maximum body width (BW)	1107	546‒1226	776 ± 211
BW/BL (%)	32	21‒43	31 ± 5
Forebody length (FO)	1749	932‒1749	1251 ± 233
FO/BL (%)	51	42‒60	51 ± 5
Oral sucker length	234	54 − 234	162 ± 35
Oral sucker width	252	98 − 252	173 ± 34
Ventral sucker length	486	276 − 486	368 ± 51
Ventral sucker width	517	319 − 557	421 ± 66
Ratio of suckers’ length	2	2‒6	3 ± 1
Ratio of suckers’ width	2	2‒4	3 ± 1
Head-collar length	98	49‒123	89 ± 17
Head-collar width	366	165‒378	271 ± 47
Pharynx length	123	77−138	98 ± 16
Pharynx width	146	61−146	88 ± 21
Ovary length	123	64−140	93 ± 19
Ovary width	108	38−118	71 ± 18
Testis 1 (posterior ovary) length	406	106−463	263 ± 81
Testis 1 (posterior ovary) width	270	80−308	154 ± 50
Testis 2 length	449	125−449	255 ± 82
Testis 2 width	147	65−268	147 ± 51
Cirrus sac length	175	59−180	99 ± 34
Cirrus sac width	135	63−204	102 ± 30
Post-testicular field length (T)	155	63−450	187 ± 79
T/Bl (%)	5	3‒13	7 ± 2
Uterus length (U)	1263	544‒1778	944 ± 300
U/Bl (%)	37	30‒50	37 ± 5
Egg length	101	76‒117	90 ± 8
Egg width	53	42‒61	51 ± 5

A multivariate analysis was conducted to evaluate the 19 morphometric characters, using a correlation model. However, the pharynx, ovary and egg size were excluded from the analysis because these structures were not visible in all specimens. The resulting multivariate data matrices were subjected to Principal Component Analysis (PCA) using PAST version 4.06b software (Hammer *et al.*, 2001). The clustering function in the programme (with default settings) was applied to generate a two-dimensional scatter plot, illustrating morphological variation among specimens, along with the corresponding percentage variances.

### Molecular and phylogenetic study

For molecular analysis, DNA was extracted from three to four specimens using the DNeasy® Blood and Tissue Kit (Qiagen, Hilden, Germany), following the manufacturer’s protocol. Amplification targeted partial sequences of four genetic markers: nuclear 18S rRNA, nuclear 28S rRNA, nuclear ITS2 and mitochondrial *COI*. These markers were selected for their proven utility in trematode systematics and molecular identification. The primers used, expected amplicon lengths and other amplification details are provided in Table S2 (Bowles *et al.*, 1992; Curran *et al.*, 2011; Routtu *et al.*, 2014; Besprozvannykh *et al.*, 2018). The forward primer for the ITS2 region was newly designed in this study, with optimisation performed prior to use. PCR amplification was conducted in a final reaction volume of 30 μL.

Reactions were performed using a T100™ thermocycler (Bio-Rad, California, USA) in a 30 µL mixture containing 15 µL of 2X i-Taq Master Mix (iNtRON Biotechnology, Gyeonggi, South Korea), 10 µM of each primer and 1 ng/µL of template DNA. Thermal cycling conditions are also provided in Table S2. PCR amplicons were visualised on a 1% agarose gel stained with SYBR Safe™ (Thermo Fisher Scientific, Massachusetts, USA). Selected PCR products were sequenced using Fast Next-Generation Sequencing (Tsingke, Beijing, China). The resulting nucleotide sequences were deposited in the NCBI database under the following accession numbers: PV383310–PV383312 (18S rRNA), PV383307–PV383309 (28S rRNA), PV682938–PV682939 (ITS2) and PV370604 (COI).

The sequences were manually inspected and edited using BioEdit version 7.2.5 (Hall, 1999). Phylogenetic analysis was conducted using maximum likelihood (ML) in MEGA-12 (Tamura *et al.*, 2013), with sequence alignment performed by ClustalX 2.1 (Thompson *et al.*, 2002). Best-fit nucleotide substitution models were determined for each marker: the Kimura 2-parameter (K2) model with a discrete gamma distribution (+G) for 28S rRNA, 18S rRNA and ITS2, and the Hasegawa-Kishino-Yano (HKY) model with a discrete gamma distribution (+G) and an additional parameter accounting for a proportion of invariable sites (+I) for *COI*. Phylogenetic trees were constructed using 1000 bootstrap replicates to ensure analytical robustness (Hall, 1999; Thompson *et al.*, 2002; Tamura *et al.*, 2013). Bayesian Inference (BI) analysis was conducted using MrBayes 3.2 (Huelsenbeck and Ronquist, 2001) using four Markov Chain Monte Carlo (MCMC) chains for 1 000 000 generations with a sampling frequency of every 100 generations. Bayesian probability values were calculated after discarding the initial 25% of trees as ‘burn-in.’ The *COI*, ITS2, 28S rRNA and 18S rRNA analyses incorporated sequences from all available species within Echinochasmidae. *Fasciola hepatica* was used as the outgroup for each marker. All sequences were retrieved from GenBank (see Figure 5). Genetic distances were calculated in MEGA-12 (Tamura *et al.*, 2013), using pairwise distance (p-distance) to assess genetic differences between species, between genera and within species.

## Results

**Taxonomy**

Phylum: Platyhelminthes Minot, 1876

Class: Trematoda Rudolphi, 1808

Order: Plagiorchiida La Rue, 1957

Family: Echinochasmidae (Odhner, 1910)

***Paratestophis* gen. nov.**

**ZooBank LSID**: urn:lsid:zoobank.org:act:FEDF5B2E-DC3D-45A7-96D9-436A7872A412

### Diagnosis

Body elongated, expanded posteriorly to ventral sucker, widest at the posterior end where testes located. Forebody narrow, forming neck-like region, giving overall bottle-shaped appearance. Collar well developed with ventrolateral edges curved medially; collar spines 22, in single row with dorsal and large ventral interruptions. Suckers well-developed; oral sucker spherical; ventral sucker large and round, located in the second quarter of body, served as a point of transition between narrow forebody and widened hindbody, clearly larger than oral sucker. Prepharynx long; pharynx small; oesophagusesophagus long. Hair-like tegumental spines, long and thin, similar length to collar spines, distributed randomly and sparsely all over body, higher density above testes, rarely in pairs. Intestinal caeca blind, bifurcated just anterior to ventral sucker and extend to posterior end, being equal in length. Testes parallel, oval or lobed, located at the posterior end of the body. Cirrus sac slightly oval, located between the intestinal bifurcation and the ventral sucker. Internal seminal vesicle saccular, bipartite. Pars prostatica indistinct. Cirrus short, within cirrus sac, anterior to ventral sucker. Genital pore anterior to ventral sucker. Ovary spherical or oval. Mehlis’ gland inconspicuous, anterior to testes. Seminal receptacle saccular, between or anterior to testes. Uterus long, from posterior margin of ventral sucker to between testes, fully filled with eggs. Eggs oval, numerous, relatively large. Post-testicular field short. Metraterm thin walled. Vitelline fields non-confluent on either side. Vitelline glands lateral, extended along intestinal caeca, originated at the level of posterior margin of the ventral sucker, terminated at the level of the anterior margin or middle of the testes; follicles small. Excretory vesicle V-shaped, occupied the post-testicular field; stem unchambered; pore terminal. Found in the large intestine of snakes.

**Type-species**: *Paratestophis gelicolus* sp. nov.

**Other species**: N/A

**ZooBank LSID**: urn:lsid:zoobank.org:act:55DF3C46-4065-469D-BB05-E1CDF07FAD7E

### Etymology

*Paratestophis* is derived from ‘para’ (Greek, meaning ‘parallel’), ‘test’ (from Latin testis, ‘testicle’) and ‘ophis’ (Greek, meaning ‘snake’). The name refers to the characteristic parallel arrangement of the testes and the species’ exclusive parasitism in snakes (para- + test- + ophis). The specific epithet *gelicolus* combines ‘gelum’ (Latin, ‘jelly’) with the suffix -colus, alluding to the worm’s bottle-like body shape, which resembles a jelly-cola candy.

**Remarks**: Based on the available samples, *Paratestophis* gen. nov. (Charoennitiwat *et al.*, 2025) possesses a collar that lacks a ventral connection ridge, with collar spines arranged in a dorsally interrupted row due to a mid-dorsal depression. These characteristics place the genus within the family Echinochasmidae (Odhner, 1910). *Paratestophis* gen. nov. is further distinguished by vitelline fields extending anteriorly to the level of the ventral sucker, setting it apart from *Dissurus* Verma, 1936, and *Stephanoprora* Odhner, 1902, in which the vitelline fields are confined to the hind body, with anterior limits at the level of the ovary or anterior testis.

The collar of *Paratestophis* gen. nov. is distinct, featuring a single row of collar spines that is dorsally interrupted, along with two angular spines on each ventral lappet. Furthermore, its internal seminal vesicle is saccular and bipartite. These traits differentiate *Paratestophis* gen. nov. from *Mehrastomum* Salsena, 1959, *Microparyphium* Dietz, 1909, and *Pulchrosomoides* Freitas & Lent, 1937. Most notably, *Paratestophis* gen. nov. exhibits a parallel alignment of the testes and primarily parasitises reptiles, particularly snakes, distinguishing it from the closely related *Echinochasmus* Dietz, 1909, *Saakotrema* Skrjabin & Bashkirova, 1956, and all other known genera within Echinochasmidae (Odhner, 1910) to date.

***Paratestophis gelicolus*** Charoennitiwat, Viriyautsahakul, & Ratnarathorn, 2025., gen. nov., sp. nov.

**Type-host**: *E. enhydris* (Schneider, 1799)

**Type-locality**: Aquatic areas such as lakes, ponds and swamps in Nakhon Si Thammarat (e.g. Pak Phraek [8°14′56.04′′ N, 100°13′18.96′′ E], Thung Song [8°17′18.6′ N, 99°29′42.6′′ E] District) and neighbouring provinces in southern Thailand (e.g. Songkhla Lake [7°12′34.2′′ N, 100°27′43.56′′ E]) served as collection sites. However, the exact coordinates of individual hosts were not documented.

**Abundance and prevalence**: Nakhon Si Thammarat and neighbouring provinces in southern Thailand: 56 parasite specimens were recovered from 25 infected out of 106 examined snakes (24%).

**Parasite intensity**: 1–12 worms, mean approximately 2

**Site in host**: Large intestine

**Deposited Material**: 30 permanently mounted individuals including one holotype (MUMNH-TRE0001) and three paratypes (MUMNH-TRE0002–0004).

**Representative DNA sequences**: 18S rRNA, three sequences (three submitted to GenBank, PV383310–PV383312); 28S rRNA, three sequences (three submitted to GenBank, PV383307–PV383309); ITS2 rDNA, two sequences (both submitted to GenBank, PV682938–PV682939 (ITS2); COI, one sequence (submitted to GenBank, PV370604).

### Etymology

The specific epithet *gelicolus* originates from ‘gelum’, meaning jelly, and ‘cola’ referring to the cola soft drink. This name is inspired by the species’ unique body shape, which closely resembles the bottle-like appearance of jelly-cola candies (Figure 1C). We propose the colloquial English name for this trematode as the ‘Jelly Cola fluke’.

### General description

**Figure 3.**
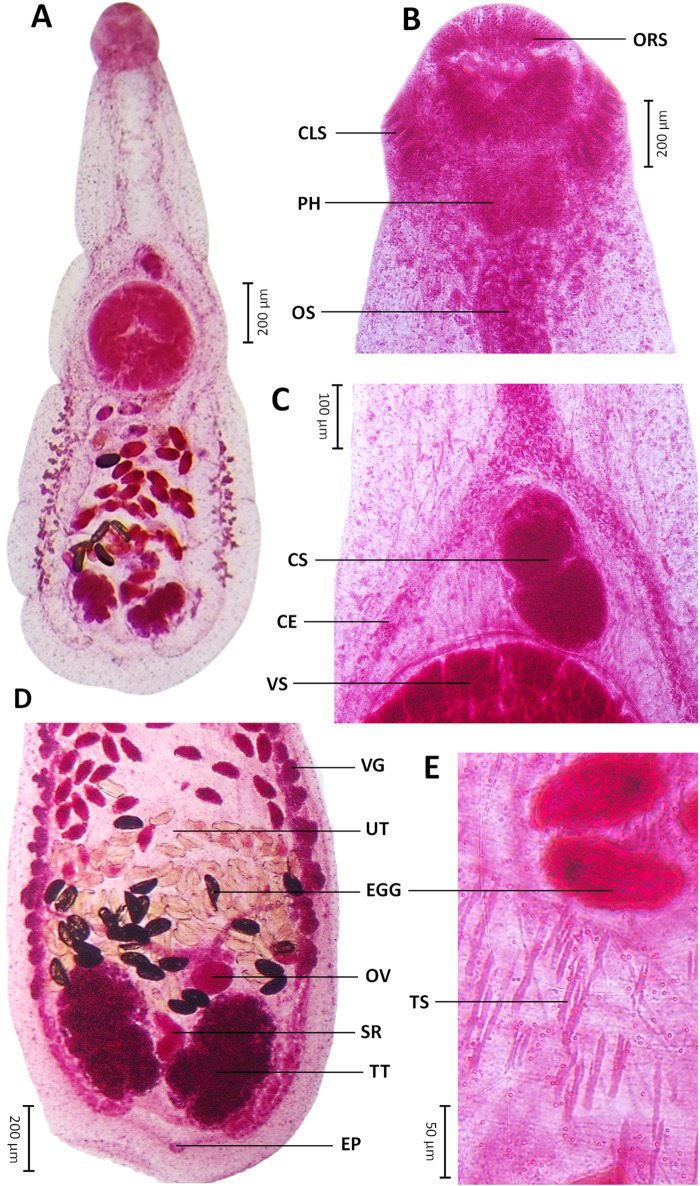
Permanent slides (acetocarmine dye) of *Paratestophis gelicolus* gen. nov., sp. nov.: (A) Entire body, ventral view; (B) Anterior region, ventral view; (C) Mid-body region, ventral view; (D) Posterior region showing internal structures and organs, ventral view; and (E) Magnified view showing tegumental spines. CE, caecum; CLS, collar spine; CS, cirrus sac; EGG, eggs; EP, excretory pore; ORS, oral sucker; OS, oesophagus; OV, ovary; PH, pharynx; SR, seminal receptacle; TS, tegumental spine; TT, testis; UT, uterus; VG, vitelline gland; VS, ventral sucker.

Based on 30 parasitic adult specimens (Table 1 and S1): Body elongated with total body length (BL) of 2496 ± 545 (1653–3523) and expanded posteriorly to ventral sucker with maximum width (BW) 776 ± 211 (546‒1226) (BW/BL = 32%) at level where testes located. Forebody narrow, forming neck-like region 1251 ± 233 (932‒1749) (Forebody length/BL = 51%), giving overall bottle-shaped appearance (Figures 1A, 1C, 2A, and 3A). Collar well developed 89 ± 17 (49–123) × 271 ± 47 (165–378), with ventrolateral edges curved medially; collar spines 22, single row with dorsal and large ventral interruptions (Figures 1B, 2B–D, and 3B). Suckers well-developed. Oral sucker spherical, 162 ± 35 (54–234) × 173 ± 34 (98–252). Ventral sucker large, round, in second quarter of body, 368 ± 51 (276–486) × 421 ± 66 (319–557), served as a point of transition between narrow forebody and widened hindbody. Ratio of ventral sucker to oral sucker length 2:1, width 3:1. Prepharynx long. Pharynx 98 ± 16 (77–138) × 88 ± 21 (61–146). Oesophagus long. Tegumental (dot-like) spines with hair-like, long, thin structure at apex, similar length to collar spines, distributed randomly and sparsely all over body, higher density above testes, rarely in pair (Figures 2F–G and 3E).

Intestinal caeca blind, bifurcated just anterior to ventral sucker and extend to posterior end, being equal in length. Testes parallel, oval or lobed (2–5 lobes, mostly 3), one side posterior to ovary 263 ± 81 (106–463) × 154 ± 50 (80–308), another 255 ± 82 (125–449) × 147 ± 51 (65–268), both at posterior end of body (Figure 3D). Cirrus sac slightly oval, 99 ± 34 (59–180) × 102 ± 30 (63–204), between intestinal bifurcation and ventral sucker (Figure 3C). Internal seminal vesicle saccular, bipartite. Pars prostatica indistinct. Cirrus short, within cirrus sac, anterior to ventral sucker. Genital pore anterior to ventral sucker (Figure 2E). Ovary spherical or oval, 93 ± 19 (64–140) × 71 ± 18 (38–118), close anterior to one testis. Mehlis’ gland inconspicuous, anterior to testes. Uterine seminal receptacle between or anterior to testes. Uterus long, from posterior margin of ventral sucker to between testes, 944 ± 300 (544–1778) (*U* = 37%) fully filled with eggs. Egg oval, numerous, relatively large 90 ± 8 (76–117) × 51 ± 5 (42–61). Post-testicular field short, 187 ± 79 (63–450) (*T* = 7%). Metraterm thin walled. Vitelline fields non-confluent on either side. Vitelline glands lateral, extended along intestinal caeca, originated at level of posterior margin of ventral sucker, terminated at level of anterior margin or middle of testes, follicles small. Excretory vesicle V-shaped, occupied post-testicular field, stem unchambered, pore terminal (Figures 1A, 2H).

**Remarks**: Adult specimens of *Paratestophis gelicolus* gen. nov., sp. nov., a new species of echinostome, were collected from 25 *E. enhydris* in Nakhon Si Thammarat and adjacent provinces in southern Thailand. The key diagnostic characteristics of the specimens include the bottle-shaped body, the presence of 22 collar spines, the parallel arrangement of the testes, and the use of snakes as the definitive host, which together justify their placement in the new genus *Paratestophis* gen. nov.

### Type materials

A parasitic specimen, designated as the holotype (Voucher No: MUMNH-TRE0001; specimen code: SN165_Holotype), along with three paratype specimens (Voucher No: MUMNH-TRE0002–0004; specimen codes: SN097_Paratype, SN162A_Paratype, SN162B_Paratype), has been deposited at the Mahidol University Museum of Natural History. All specimens were recovered from the large intestine of rainbow water snakes (*E. enhydris*). The snake host for the holotype (Project ID: SN165; AASL ID: AAS182 [Co-Ee-106]), and SN162A_Paratype and SN162B_Paratype (Project ID: SN162; AASL ID: AAS179 [Co-Ee-103]) were collected on 7 February 2025, while the host for the SN097_Paratype (Project ID: SN097; AASL ID: AAS114 [Co-Ee-070]) were collected on 27 September 2023. The trematode specimens were obtained by author team from the Department of Helminthology, Faculty of Tropical Medicine, Mahidol University. Morphometric measurements for the holotype are detailed (Table 1), with its morphological characteristics aligning with the general description.

### Variation

**Figure 4.**
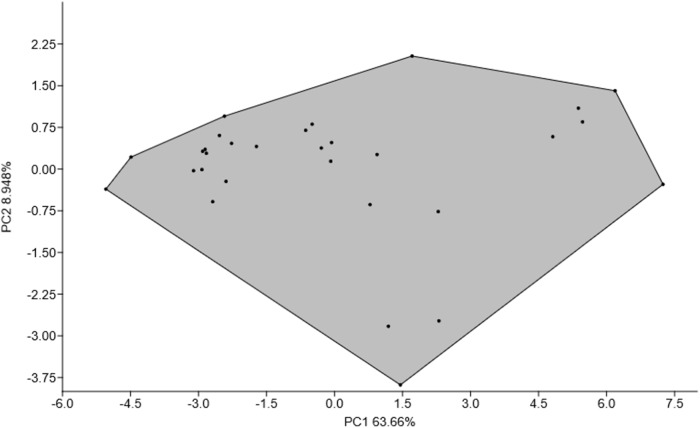
Principal Component Analysis (PCA) of *Paratestophis gelicolus* gen. nov., sp. nov. specimens. The PCA was conducted using 19 morphological characters, explaining 72.608% of the total variance. Black dots and lines represent individual specimens.

The PCA two-dimensional plot of PC1 and PC2 illustrated no clear separation or morphological clustering, indicating no population or diversification among the 30 examined *P. gelicolus* gen. nov., sp. nov. (Figure 4). The PCA accounting for 72.608% of the total variance further supported the homogeneity of the examined *P. gelicolus* gen. nov., sp. nov. samples with a marked decline in eigenvalues from PC1 (63.660%) to PC2 (8.948%), and from PC2 to PC3 (7.386%).

### Genetic characterisation and phylogenetic position

**Figure 5.**
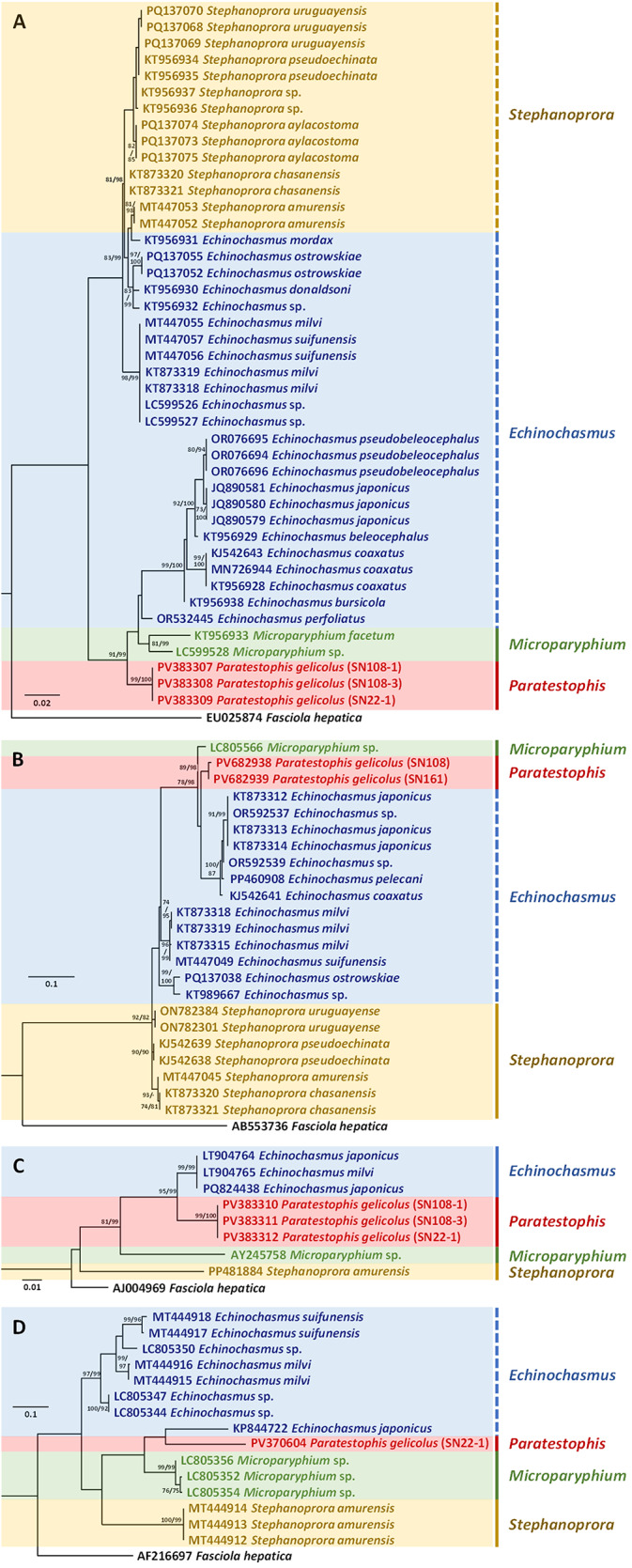
Phylogenetic analyses of available trematode species sequences from the family Echinochasmidae, with *Fasciola hepatica* as an outgroup, based on the 28S rRNA (A), ITS2 (B), 18S rRNA (C), and *COI* (D) genes. Analyses were performed using the maximum likelihood method in MEGA-12. Branch length scale bars indicate the number of substitutions per site, and node values represent bootstrap support from Maximum Likelihood and Bayesian Inference. Coloured markers indicate the different genera of echinochasmids retrieved from GenBank. *Paratestophis gelicolus* gen. nov., sp. nov., Identified in this study, is highlighted in red (font/box). Solid lines denote monophyletic groups, while dashed lines indicate non-monophyletic groupings.

Phylogenetic analyses of the four genes (18S rRNA, ITS2, 28S rRNA and *COI*) revealed that the specimens of *Paratestophis gelicolus* gen. nov., sp. nov. formed a distinct clade, clearly separated from other echinochasmid genera (Figures 5A–D). The genetic distance matrix provided further support for *Paratestophis* gen. nov. as a novel genus, with genetic divergence (between genera) ranging from 2.7% to 8.8% for 18S, 3.6% to 10.1% for ITS2, 3.6% to 4.9% for 28S, and 14.5% to 19.4% for *COI*. In terms of phylogenetic relationships, all phylogenetic trees consistently indicated that *Paratestophis* gen. nov. is closely related to *Echinochasmus* and *Microparyphium*, both of which primarily parasitise different vertebrate hosts (i.e. mammals and birds) compared to *Paratestophis* gen. nov. Although *Stephanoprora*, another member of Echinochasmidae, utilises reptiles (e.g. crocodiles) as definitive hosts, phylogenetic analyses indicated a distant relationship between *Paratestophis* gen. nov. and *Stephanoprora*. Interestingly, the *COI* analysis revealed a close sequence similarity between *P. gelicolus* gen. nov., sp. nov. and *Echinochasmus japonicus*, where genus *Echinochasmus* was not monophyletic (Figure 5D). With the 18S and 28S genetic markers, no intraspecies genetic variation was observed among the *Paratestophis gelicolus* gen. nov., sp. nov. specimens, while a 0.8% genetic difference was obtained for the ITS2 region.

### Natural history

*Paratestophis gelicolus* gen. nov., sp. nov. exhibits a moderate prevalence in its host, the rainbow water snake, *E. enhydris*, with 25 examined specimens testing positive for infection out of 106 snakes (prevalence = 24%). This trematode is the only helminth species infecting the large intestine of the snakes, which were captured in Nakhon Si Thammarat (e.g. Pak Phraek, Thung Song District) and neighbouring provinces in southern Thailand (e.g. Songkhla). This finding is particularly significant as it represents the first identification of a parasite from the genus *Paratestophis* gen. nov. and an unusual case of reptile infection by Echinochasmidae. In addition, this discovery marks the first identification of Echinochasmidae in snakes. Currently, only adult hermaphroditic parasites have been documented in the host, with no investigation yet conducted into associated lesions or other aspects of its life cycle.

### Updated key to genera of the family Echinochasmidae

Phylogenetic analyses based on the 28S rRNA, ITS2, 18S rRNA, and *COI* genetic markers confirm that *Paratestophis* gen. nov. belongs to the family Echinochasmidae and forms a well-supported genetically distinct lineage (Figure 5). All four markers reinforce the taxonomic validity of the genus, clearly separating *Paratestophis* gen. nov. from other genera within the family, including *Echinochasmus, Stephanoprora* and *Microparyphium* (Figure 5). Across all genetic datasets, *Paratestophis* gen. nov. consistently forms a monophyletic clade alongside *Microparyphium* and selected species of *Echinochasmus*, suggesting a close evolutionary relationship among these three genera. Non-monophyly of *Echinochasmus* was observed in the phylogenies based on 28S, ITS2 and *COI* markers, consistent with previous studies (Tkach *et al.*, 2016; Kalinina *et al.*, 2023; Islas-Ortega *et al.*, 2024). *Echinochasmus* remains the most genetically represented genus within the family, with 11 and 6 species available for the 28S and ITS2 markers, respectively. In contrast, genetic data for *Stephanoprora* and *Microparyphium* are comparatively limited. Furthermore, due to the absence of molecular data for several other genera within Echinochasmidae (e.g. *Dissurus, Mehrastomum* and *Pulchrosomoides*), the overall phylogenetic relationships within the family remain partially unresolved.

Morphologically, *Paratestophis* gen. nov. is distinguished from all previously described genera within Echinochasmidae by the first recorded parallel arrangement of testes, a feature not previously observed in the family. In addition, this genus exhibits a unique host association, having been found parasitising snakes, whereas other members of Echinochasmidae predominantly parasitise birds or mammals. These distinctive morphological and ecological traits provide strong support for the recognition of *Paratestophis* gen. nov. as a novel genus. Based on morphological and molecular evidence, the family Echinochasmidae Odhner, 1911, traditionally comprised seven genera–*Dissurus* Verma, 1936; *Echinochasmus* Dietz, 1909; *Mehrastomum* Saksena, 1959; *Microparyphium* Dietz, 1909; *Pulchrosomoides* Freitas & Lent, 1937; *Saakotrema* Skrjabin & Bashkirova, 1956; and *Stephanoprora* Odhner, 1902 (Tkach *et al.*, 2016; Islas-Ortega *et al.*, 2024)–now includes the newly identified genus *Paratestophis* gen. nov. as the eighth genus. A diagnostic key to the genera of Echinochasmidae is provided herein. This key is an amended version of Kostadinova (2005), and for the known genera, the characters are taken from Keys to the Trematoda, Vol. 2 (Jones *et al.*, 2005).

### Key to genera: Family echinochasmidae (odhner 1910)

1a.: Vitelline fields confined to hind body, with anterior limits at level of ovary or anterior testis _____________________________**2**
1b.: Vitelline fields extend anteriorly to level of ventral sucker of further into forebody ________**3**
2a.: Body markedly elongate and slender (BW < 10%); collar-spines 24, with no special angle spine groups; testes fairly close to posterior extremity (*T* < 15%); ovary equatorial; uterus long (*U* > 30%) _____________________________***Dissurus* Verma, 1936**
2b.: Body elongate or elongate-oval (BW = 10–13%); collar-spines 22 (26 in type-species only); two angle spines present on each ventral lappet; testes equatorial, just pre- or just post-equatorial (*T* = 30–55%); ovary pre-equatorial; uterus short (*U* < 15%) __________________________***Stephanoprora* Odhner, 1902**
3a.: Collar poorly developed; ventrolateral collar-spines in double row; laterodorsal spines in single row; dorsal interruption wide; no special angle spine groups; internal seminal vesicle simple, with saccular posterior and tubular anterior regions _______________________**4**
3b.: Collar distinct; collar-spines all in single row; dorsal interruption narrow; two angle spines present on each ventral lappet; internal seminal vesicle saccular, bipartite __________________________**6**
4a.: Testes oblique; post-testicular field short (*T* < 20%); ovary between third and last quarter of body; vitelline fields between intestinal bifurcation and posterior extremity, approach median line in forebody _____________________***Mehrastomum* Salsena, 1959**
4b.: Testes tandem; post-testicular field long (*T* > 25%); ovary equatorial of jus pre-equatorial; vitelline fields between level of ventral sucker, or anterior hind body, and posterior extremity __________________**5**
5a.: Collar-spines 20–24, sharply pointed; in birds _______________________***Microparyphium* Dietz, 1909**
5b.: Collar-spines >50, blunt; in reptiles ________________***Pulchrosomoides* Freitas & Lent, 1937**
6a.: Testes tandem or oblique, in birds and mammals _________________________**7**
6b.: Testes parallel, in reptiles__________________***Paratestophis*** Charoennitiwat, Viriyautsahakul, & Ratnarathorn, 2025
7a.: Entire tegument armed with large spines (60–80% of maximum collar-spine length); uroproct present; uterus long, with descending limb forming few loops posteriorly to testes ___________________________***Saakotrema* Skrjabin & Bashkirova, 1956**
7b.: Tegumental spines dense in forebody only, smaller (30–50% of maximum collar-spine length); caeca blind _______________***Echinochasmus* Dietz, 1909**

## Discussion

A new genus and species of echinostome digenean, *Paratestophis gelicolus* gen. nov., sp. nov., was discovered in the large intestine of the rainbow water snake, *E. enhydris*, in southern Thailand. Genetic analyses based on nuclear and mitochondrial genetic markers indicate that this new species forms a distinct lineage within the family Echinochasmidae. Although differences in the phylogenetic placement of *Paratestophis* gen. nov. were observed between the nuclear and mitochondrial genetic markers, genetic differences and morphological characteristics clearly distinguish *P. gelicolus* gen. nov., sp. nov. from all previously described genera within the family. Discrepancies between nuclear and mitochondrial genes in digenean classification have been previously documented (e.g. Brabec *et al.*, 2015; Locke *et al.*, 2018; Thaenkham *et al.*, 2022). These findings reinforce that widely used nuclear markers provide greater taxonomic resolution for distinguishing genera within Echinochasmidae (e.g. Islas-Ortega *et al.*, 2024). Additionally, the necessity of integrating both morphological and molecular data for robust and accurate taxonomic identification is emphasised (Thaenkham *et al.*, 2022; Charoennitiwat *et al.*, 2023, 2024a, 2024b, 2025).

As members of Echinochasmidae typically parasitise mammals and birds in their adult stage (Tkach *et al.*, 2016), with only a few cases previously reported in other reptiles (Platt 2006; Cajiao-Mora *et al.*, 2025), the discovery of *P. gelicolus* gen. nov., sp. nov. also marks the first identification of an echinostome infecting snakes. The presence of adult specimens suggests that the rainbow water snake serves as the definitive host, likely infected through the ingestion of parasitised prey.

The rainbow water snake, *E. enhydris* (Schneider 1799), is a common and widely distributed snake across the Oriental regions and Thailand (Ratnarathorn *et al.*, 2024). This species primarily preys on fishes and occasionally small amphibians (Cox *et al.*, 2012), which this digenean species might parasitise the snake via their food, potentially serving as intermediate hosts and definitive hosts, respectively for the completion of a parasite’s life cycle (Vattakaven *et al.*, 2016; Lopez and Duffy 2021). The identification of parasitic infections in snakes underscores the importance of wildlife health monitoring and management, particularly in the context of exotic pet ownership and interactions with wild animals (e.g. Charoennitiwat *et al.*, 2024b).

Although *P. gelicolus* gen. nov., sp. nov. is the only helminth infecting the large intestine of *E. enhydris*, it co-infected the host alongside at least four other helminth species, including *Tanqua siamensis, Encyclometra bungara*, and trematode species currently under examination. Each of these occupies a distinct organ and appears to be the sole representative therein (Chan *et al.*, 2024; Charoennitiwat *et al.*, 2024b). Multiparasitism is common in tropical regions with high parasite diversity and often leads to interspecific competition for limited space and resources. Competition may be disadvantageous for weaker species, frequently resulting in reduced body size and fecundity (Dezfuli *et al.*, 2002; Lagrue and Poulin, 2008; Vaumourin *et al.*, 2015). Thus, the organ-specific distribution of helminths in *E. enhydris* may reflect a co-evolutionary strategy to minimise interspecific competition by restricting each species to a specific organ best suited to its ecological niche (Poulin, 2001). Notably, *T. siamensis* and *E. bungara* exhibit significantly higher infection intensity and prevalence than *P. gelicolus* gen. nov., sp. nov., suggesting they may be stronger competitors (Chan *et al.*, 2024; Charoennitiwat *et al.*, 2024b). Future studies should investigate the overall parasite community structure and interactions within *E. enhydris* to further elucidate competitive dynamics (e.g. Ratnarathorn *et al.*, 2025).

With the inclusion of *Paratestophis* gen. nov., the family Echinochasmidae now comprises eight genera, highlighting the growing diversity within the family. However, the full taxonomic placement of genera within Echinochasmidae remains incomplete due to the lack of genetic sequences for many identified genera. To date, genetic data–particularly the 28S rRNA gene, which is the most commonly used for the systematic study of trematodes (Thaenkham *et al.*, 2022; Islas-Ortega *et al.*, 2024)–are available for only four genera. This limited sampling suggested that additional taxonomic revisions, incorporating further genetic markers (Thaenkham *et al.*, 2022), may be necessary to clarify relationships within the family. The observed genetic divergence within *Echinochasmus* indicates that further subdivision of this genus may be necessary, as the non-monophyly of *Echinochasmus* and *Stephanoprora* was observed. These findings highlight the need for further molecular and morphological investigations to refine the systematic framework of Echinochasmidae.

*Echinochasmus* and *Stephanoprora* are among the most medically and veterinary important genera in Echinochasmidae, having been implicated in zoonotic infections (Sayasone *et al.*, 2009; Tkach *et al.*, 2016; Islas-Ortega *et al.*, 2024). This medical significance has likely driven more extensive research on these genera, resulting in a greater accumulation of genetic and morphological data compared to other genera in the family. To establish a more comprehensive evolutionary framework, future research should focus on expanding genetic data for lesser-studied genera, conducting morphological reassessments of existing taxa, and exploring host-specific adaptations. Broader taxon sampling and more extensive molecular data are essential to resolve the phylogenetic relationships within Echinochasmidae and clarify species boundaries.

In conclusion, the discovery and description of *Paratestophis gelicolus* gen. nov., sp. nov., a new species of Echinochasmidae, is reported in this study from the rainbow water snake (*E. enhydris*) in southern Thailand. Genetic analysis suggests that this trematode species represents a new genus, *Paratestophis* gen. nov., with additional support from morphological examination, which reveals that *Paratestophis* gen. nov. exhibits a parallel alignment of testes and 22 collar spines–clearly distinguishing it from all other described genera within the family. This discovery also marks the first record of Echinochasmidae in snakes, further differentiating it from other genera that primarily infect birds and mammals. As a newly described genus, the systematic framework of Echinochasmidae has been updated to include *Paratestophis* gen. nov., increasing the number of genera within the family to eight. However, reassessment incorporating both genetic and morphological data is recommended on lesser-studied genera to resolve the remaining taxonomic complications in the family. Additionally, since *P. gelicolus* gen. nov., sp. nov. co-infects *E. enhydris* alongside multiple other helminth species, future studies could aim to examine these infections in a broader evolutionary and ecological context, focusing on helminth communities or their evolutionary history within *E. enhydris*.

## Supporting information

Charoennitiwat et al. supplementary material 1 Charoennitiwat et al. supplementary material

Charoennitiwat et al. supplementary material 2 Charoennitiwat et al. supplementary material

## Data Availability

The data that support the findings of this study are available from the first and corresponding authors upon reasonable request.

## Supplementary materials

The supplementary material for this paper can be found at https://doi.org/10.1017/S0031182025100863.
