# Supplementary material for: The discovery of Paratestophis gelicolus gen. nov., sp. nov. from the rainbow water snake, Enhydris enhydris, in Thailand, with systematic update of Echinochasmidae Odhner, 1910
Source: Parasitology. 2025 Oct 16;152(13):1355–66. doi: 10.1017/S0031182025100863 (PMC12917397; doi:10.1017/S0031182025100863)
Supplement: Charoennitiwat et al. supplementary material 2 — Charoennitiwat et al. supplementary material [file S0031182025100863sup002.docx]

**Table S2:** Specific primers, thermal cycling conditions, and PCR amplification details for the molecular investigation of *Paratestophis gelicolus* gen. nov., sp. nov.

| **Gene** | **Primer** | **Primer sequence** | **Length (bp)** | **Denaturation** | **Extension** | **Reference** |
| --- | --- | --- | --- | --- | --- | --- |
| 18S rRNA | C | 5′-ATGGCTCATTAAATCAGCTAT-3′ | 800  (partial) | 94 °C_5 min, 35 cycles of 94 °C_1 min, 53 °C_1 min, 72 °C_2 min | 72 °C_10 min | Routtu *et al*., 2014 |
|  | Arev | 5′-TGCTTTGAGCACTCAAATTTG-3′ |  |  |  |  |
| 28S rRNA | Digl2 | 5′-AAGCATATCACTAAGCGG–3′ | 1,200  (partial) | 94 °C_3 min, 34 cycles of 94 °C_45 sec, 54 °C_45 sec 72 °C_2 min | 72 °C_10 min | Curran *et al*., 2011 |
|  | 1500R | 5′-GCTATCCTGAGGGAAACTTCG-3′ |  |  |  |  |
| ITS2 | 5.8SF-Echinochasmidae | 5′-CTGCTTTGAACATCGACATC-3′ | 995 | 94 °C_5 min, 34 cycles of 95 °C_30 sec, 55 °C_30 sec, 72 °C_2 min | 72 °C_5 min | Besprozvannykh *et al*., 2018 |
|  | 28S4R | 5′-TATTTAGCCTTG GATGGAGTTTACC-3′ | (partial) |  |  |  |
| *COI* | JB3 | 5′-TTTTTTGGGCATCCTGAGGTTTAT-3′ | 440 | 95 °C_5 min, 34 cycles of 95 °C_1 min, 52 °C_1 min, 72 °C_1 min | 72 °C_5 min | Bowles *et al*., 1992 |
|  | JB4.5 | 5′-TAAAGAAAGAACATAATGAA AATG-3′ | (partial) |  |  |  |
